# Supplementary material for: Full Skin Equivalent Models for Simulation of Burn Wound Healing, Exploring Skin Regeneration and Cytokine Response
Source: J Funct Biomater. 2023 Jan 4;14(1):29. doi: 10.3390/jfb14010029 (PMC9864292; doi:10.3390/jfb14010029)
Supplement: Supplementary file 1 [file jfb-14-00029-s001.zip › jfb-2116469-supplementary.pdf]

SUPPLEMENTARY TABLES

Supplementary Table S1. Culture medium for cells and full skin equivalents.

Medium	Contents
**Culture medium**	Dulbecco's Modified Eagle's Medium (DMEM), 10% fetal calf serum (Fetalclone III, Logan, UT); 1% 200 mM glutamine, antibiotics (100 IU/mL penicillin, 100 mg/mL streptomycin; Invitrogen)
**FSE I medium**	DMEM/Ham's F12 Nutmix (3:1) (Invitrogen, Paisley, UK), 5% fetal calf serum (Fetalclone III, Logan, UT), 1 mM hydrocortisone, 1 mM isoproterenol, 0.1 mM insulin, a lipid supplement (25 mM palmitic acid, 15 mM linoleic acid, 7 mM arachidonic acid, and 24 mM bovine serum albumin) (all Sigma-Aldrich, St. Louis, MO), antibiotics (100 IU/mL penicillin, 100 mg/mL streptomycin; Invitrogen)
**FSE II medium**	DMEM/Ham's F12 Nutmix (3:1) (Invitrogen, Paisley, UK), 2% fetal calf serum (Fetalclone III, Logan, UT), 1 mM hydrocortisone, 1 mM isoproterenol, 0.1 mM insulin, 1.9 μM DL-a-tocoferol, a lipid supplement (25 mM palmitic acid, 15 mM linoleic acid, 7 mM arachidonic acid, and 24 mM bovine serum albumin) (all Sigma-Aldrich, St. Louis, MO), antibiotics (100 IU/mL penicillin, 100 mg/mL streptomycin; Invitrogen)
**FSE III medium**	DMEM/Ham's F12 Nutmix (3:1) (Invitrogen, Paisley, UK), 0.5% fetal calf serum (Fetalclone III, Logan, UT), 1 mM hydrocortisone, 1 mM isoproterenol, 0.1 mM insulin, 1.9 μM DL-a-tocoferol, 130 mg/mL ascorbic acid, a lipid supplement (25 mM palmitic acid, 15 mM linoleic acid, 7 mM arachidonic acid, and 24 mM bovine serum albumin) (all Sigma-Aldrich, St. Louis, MO), antibiotics (100 IU/mL penicillin, 100 mg/mL streptomycin; Invitrogen)

Supplementary Table S2. Antibodies used for immunohistochemistry.

Primary antibody	Clone	Host	Dilution	Manufacturer	Antigen retrieval
**Pan-cytokeratin**	PCK-26	Mouse	1:200	Sigma	Triton
**Cytokeratin 10**	Polyclonal	Rabbit	1:5000	Abcam	Citrate
**Cytokeratin 15**	EPR1614Y	Mouse	1:200	Abcam	Citrate
**Cytokeratin 17**	E3	Mouse	1:500	DAKO	Citrate
**Involucrin**	SY5	Mouse	1:250	Novocastra	Triton
**Collagen IV**	CIV 22	Mouse	1:100	DAKO	Triton
**Laminin α 5**	4C7	Mouse	1:200	DAKO	Triton
**Vimentin**	V9	Mouse	1:1000	DAKO	Triton
**α-SMA**	1A4	Mouse	1:500	DAKO	Tris/EDTA
**Ki67**	MIB1	Mouse	1:100	DAKO	Tris/EDTA
**BrdU**	IIB5	Mouse	1:200	MP Biomedicals	HCl/Borax

SUPPLEMENTARY FIGURES

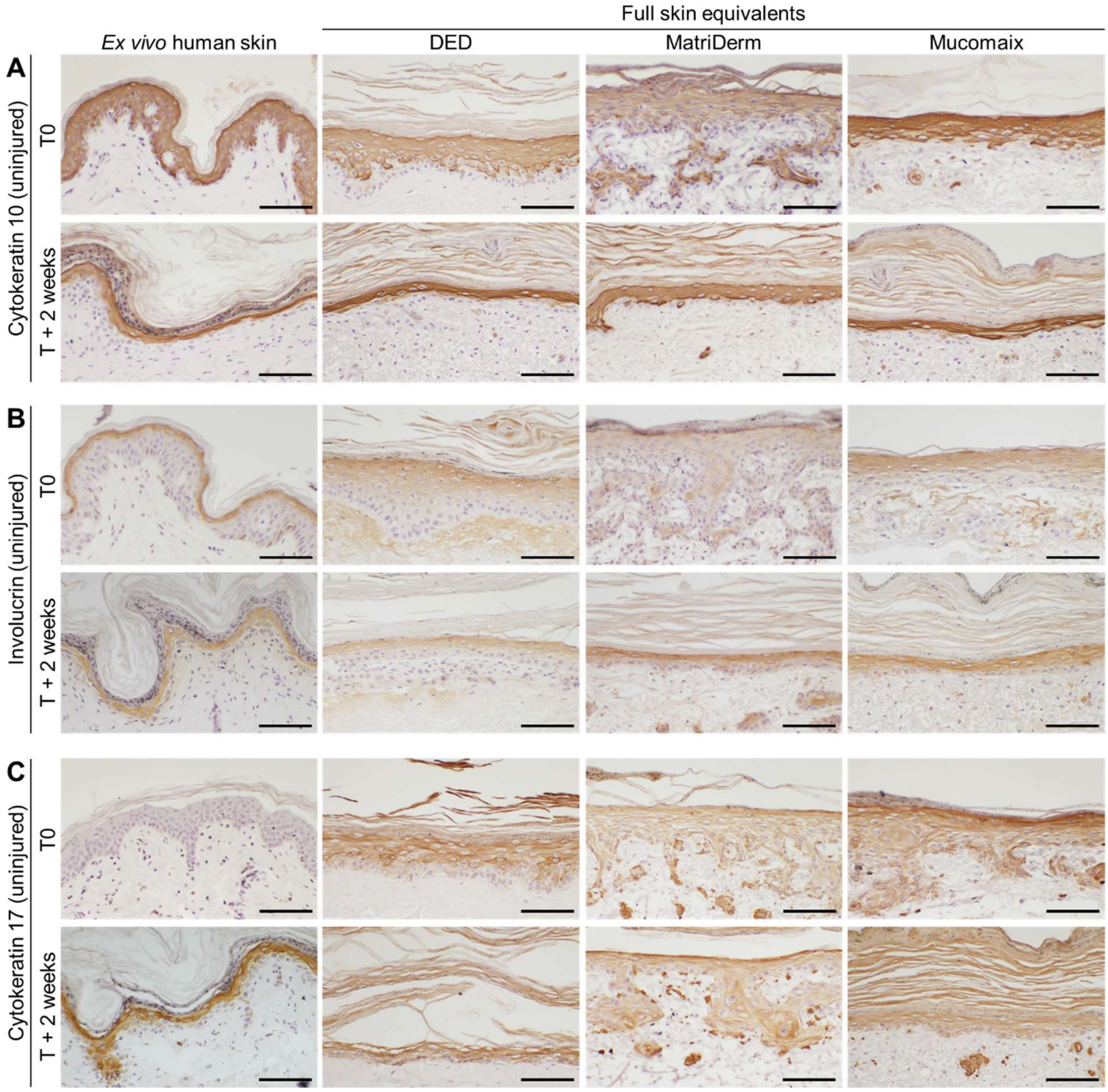

Supplementary Figure S1. Microscopic images of cytokeratin 10, involucrin and cytokeratin 17 in cultured skin models. Ex vivo human skin (left) and full skin equivalents generated from de-epidermalized dermis, MatriDerm and Mucomaix (right). Immunohistochemical **(A)** cytokeratin 10; **(B)** involucrin; **(C)** cytokeratin 17 DAB staining. Models were produced from 3 different skin donors in duplicate. For the full skin equivalent models, day 0 was after the initial 3 weeks of culture. Black scale bar = 100 μm.

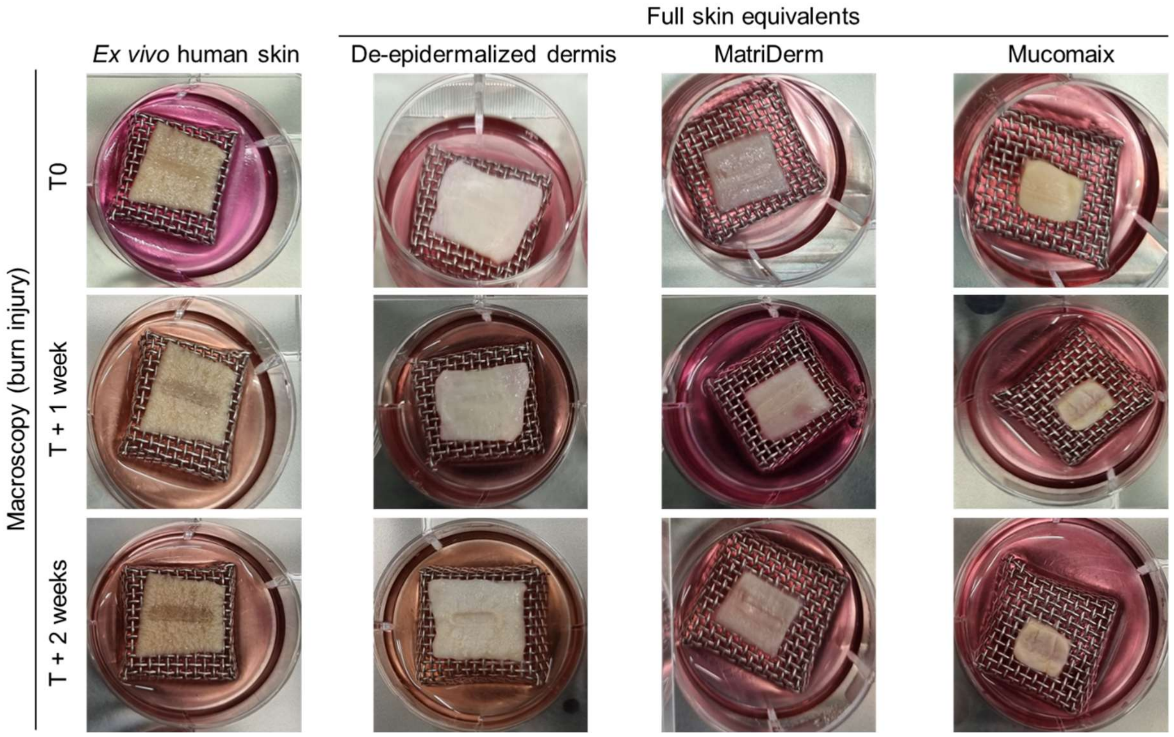

Supplementary Figure S2. Macroscopic images of burn-injured skin models. *Ex vivo* human skin (left) and full skin equivalents generated from de-epidermalized dermis, MatriDerm and Mucomaix (right). Models were produced from 3 different skin donors in duplicate. For the full skin equivalent models, day 0 was after the initial 3 weeks of culture.

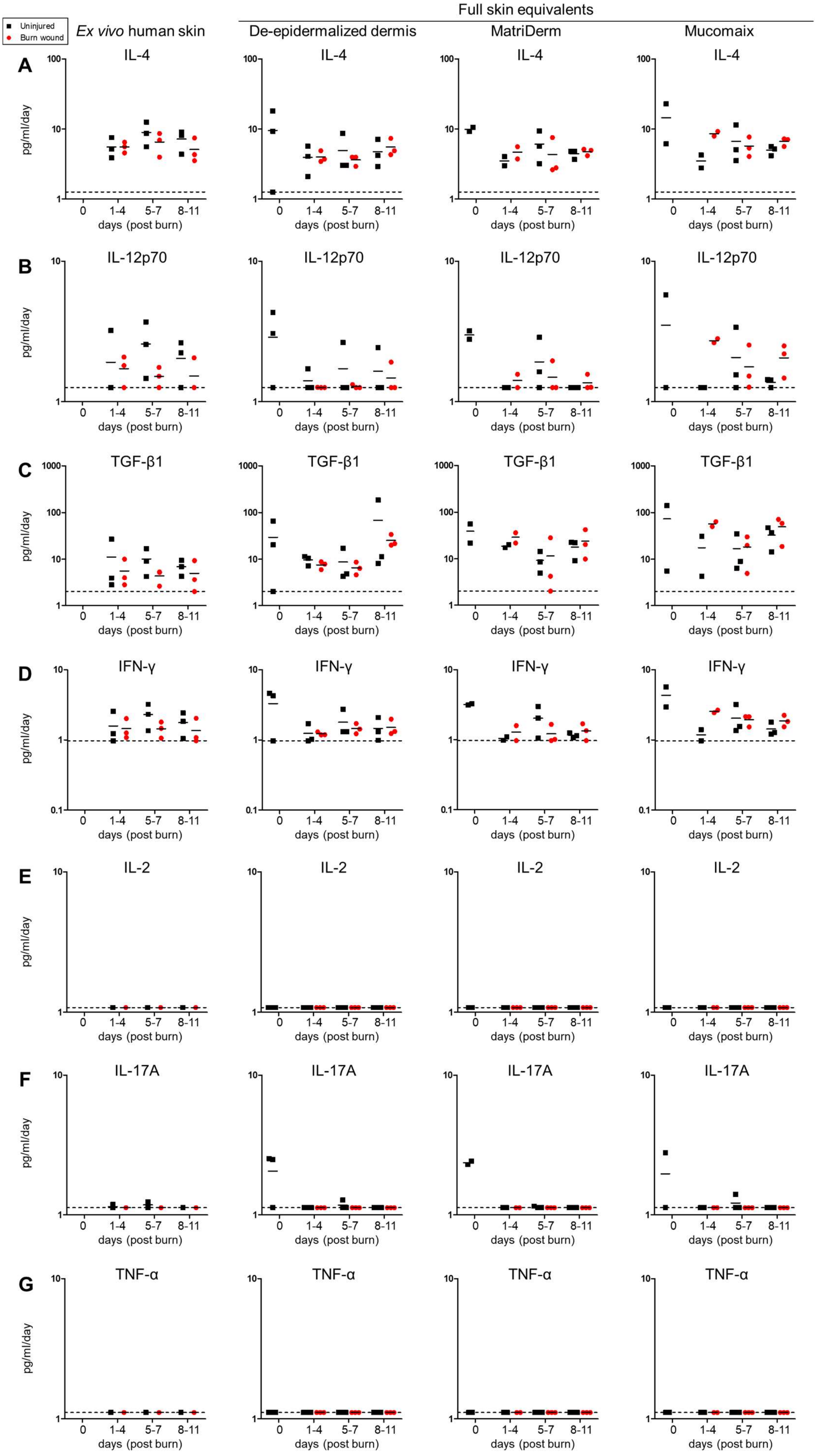
Uninjured
Burn wound
Full skin equivalents
Ex vivo human skin
De-epidermalized dermis
MatriDerm
Mucomaix
A
B
C
D
E
F
G
IL-4
IL-12p70
TGF-β1
IFN-γ
IL-2
IL-17A
TNF-α
pg/ml/day
days (post burn)
0
1-4
5-7
8-11

Supplementary Figure S3. Cytokines detected in culture medium of burn-injured and uninjured skin models. *Ex vivo* human skin (left) and full skin equivalents generated from de-epidermalized dermis, MatriDerm and Mucomaix (right). Level of **(A)** IL-4; **(B)** IL-12p70; **(C)** TGF-β1; **(D)** IFN-γ; **(E)** IL-2; **(F)** IL-17A; **(G)** TNF-α in the culture medium at T0, T + 1-4, T + 5-7 and T + 8-11 days (after burn injury). Samples from biological duplicates were pooled per donor (n = 3 donors) and re-calculated into pg/ml per day of culture to compensate for intermittent medium changes. Striped line indicates the highest or lowest level of quantification. Because ex vivo human skin models were started at T0, no levels are shown for day 0. For the full skin equivalent models, day 0 was after the initial 3 weeks of culture.
